# Prion Formation and Polyglutamine Aggregation Are Controlled by Two Classes of Genes

**DOI:** 10.1371/journal.pgen.1001386

**Published:** 2011-05-19

**Authors:** Anita L. Manogaran, Joo Y. Hong, Joan Hufana, Jens Tyedmers, Susan Lindquist, Susan W. Liebman

**Affiliations:** 1 Department of Biological Sciences, Laboratory for Molecular Biology, University of Illinois at Chicago, Chicago, Illinois, United States of America; 2 Department of Biological Sciences, University of Wisconsin-Milwaukee, Milwaukee, Wisconsin, United States of America; 3 Zentrum fuer Molekulare Biologie Heidelberg, DKFZ-ZMBH-Alliance, Universitaet Heidelberg, Heidelberg, Germany; 4 Whitehead Institute for Biomedical Research and Howard Hughes Medical Institute, Department of Biology, Massachusetts Institute of Technology, Cambridge, Massachusetts, United States of America; National Institute of Diabetes and Digestive and Kidney Diseases, United States of America

## Abstract

Prions are self-perpetuating aggregated proteins that are not limited to mammalian systems but also exist in lower eukaryotes including yeast. While much work has focused around chaperones involved in prion maintenance, including Hsp104, little is known about factors involved in the appearance of prions. *De novo* appearance of the [*PSI* ^+^] prion, which is the aggregated form of the Sup35 protein, is dramatically enhanced by transient overexpression of *SUP35* in the presence of the prion form of the Rnq1 protein, [*PIN* ^+^]. When fused to GFP and overexpressed in [*ps^−^*] [*PIN* ^+^] cells, Sup35 forms fluorescent rings, and cells with these rings bud off [*PSI* ^+^] daughters. We investigated the effects of over 400 gene deletions on this *de novo* induction of [*PSI* ^+^]. Two classes of gene deletions were identified. Class I deletions (*bug1Δ*, *bem1Δ*, *arf1Δ*, and *hog1Δ*) reduced the efficiency of [*PSI* ^+^] induction, but formed rings normally. Class II deletions (*las17Δ*, *vps5Δ*, and *sac6Δ*) inhibited both [*PSI^+^*] induction and ring formation. Furthermore, class II deletions reduced, while class I deletions enhanced, toxicity associated with the expanded glutamine repeats of the huntingtin protein exon 1 that causes Huntington's disease. This suggests that prion formation and polyglutamine aggregation involve a multi-phase process that can be inhibited at different steps.

## Author Summary

Certain proteins that exist in functional unaggregated conformers can also form self-perpetuating infectious aggregates called prions. Here we investigate factors involved in the initial switch to the prion form. *De novo* appearance of the [*PSI* ^+^] prion, which is the aggregated form of the Sup35 protein, is dramatically enhanced by overexpression of the *SUP35* gene in the presence of the prion form of the Rnq1 protein, [*PIN* ^+^]. When tagged with green fluorescent protein and transiently overexpressed in [*psi^−^*] [*PIN* ^+^] cells, Sup35 forms fluorescent rings, and cells with these rings give rise to daughter cells that are [*PSI* ^+^]. Here, we investigate factors required for this induction of [*PSI* ^+^]. Analyses of over 400 gene deletions revealed two classes that reduce [*PSI* ^+^] induction: one class forms fluorescent rings normally, and the other does not. Interestingly, the former class enhanced, while the latter class reduced, toxicity associated with the expanded polyglutamine repeats of the huntingtin protein exon 1 that causes Huntington's disease. These results suggest that prion formation and polyglutamine aggregation involve a multi-phase process that can be inhibited at different steps.

## Introduction

Prions are associated with transmissible spongiform encephalopathies, a family of neurological diseases that include Creutzfeldt-Jakob disease in humans, Scrapie in sheep, and the well-publicized “Mad Cow” disease. Transmission of prions occurs when a normally folded protein is converted to an alternate conformation that has the ability to further convert additional molecules of the normal protein into the misfolded infectious form.

Prions also exist in *Saccharomyces cerevisiae*. The well characterized cytoplasmically transferred elements [*URE3*], [*PSI* ^+^], and [*PIN* ^+^] (see reviews [1]–[4]), as well as several other recently characterized elements [5]–[9], have been identified as yeast prions.

Prions occur spontaneously in laboratory strains, although at very low frequency [10]–[12]. *De novo* appearance of prions can be facilitated by overexpression of either the whole prion protein or distinct regions that are required for propagation, called prion domains [13]–[19]. Most known yeast prion domains are glutamine (Q) and asparagine (N) rich [3], [5]–[9], [20]. In the case of *de novo* appearance of the prion form of the Sup35 translational termination factor, [*PSI* ^+^], overexpression of Sup35 or its prion domain (Sup35PD) dramatically increases the appearance of [*PSI* ^+^]. However, this increase requires either Q/N-rich domains that are simultaneously overexpressed [21], [22] or the presence of another Q/N rich prion, like the prion form of the Rnq1 protein, [*PIN^+^*] (also called [*RNQ* ^+^]; [22]–[24]).

Similar to prion strains found in mammals (reviewed in [25]), yeast prions have also been shown to exist in different conformations called “variants” [14], [26]–[30]. The introduction of *in vitro* generated Sup35 amyloid fibers into yeast not only infects the cells with the prion, proof of the “protein-only” hypothesis, but also demonstrates that distinct forms of the *in vitro* made amyloid cause the appearance of distinct variants that are heritable [31]–[32].

To further understand how prions are formed and maintained, recent studies have focused on specific host factors that affect the propagation and appearance of yeast prions. Chaperones, which are normally involved in proper protein folding, play a role in prion maintenance and appearance. Hsp104 and Sis1 break prion aggregates into smaller pieces that efficiently segregate into daughter cells, a requirement for prion transmission [33]–[41]. Deletion of the N-terminal activation domain of Hsf1, a heat shock transcription factor, prevents [*PSI* ^+^] formation, while deletion of the Hsf1 C-terminal region promotes [*PSI* ^+^] appearance [42]. Furthermore, disruption of the non-essential human Hsp110 ortholog, *SSE1,* or overexpression of *HSP82* and *HSC82* that encode members of the Hsp90 family of chaperones, dramatically reduces, but does not eliminate, the induction of [*PSI* ^+^] caused by the overexpression of Sup35PD [43].

Factors that affect [*PSI* ^+^] induction are not limited to chaperones. Deletion of actin cytoskeletal genes, such as *SLA1* or *SLA2*, reduces [*PSI* ^+^] induction [44], suggesting that the actin cytoskeleton may play a role in prion appearance. Deletion of the ubiquitin-conjugating enzyme, Ubc4, enhances the *de novo* appearance of [*PSI* ^+^] [45], and exposure to environmental stress can also alter the frequency of [*PSI* ^+^] appearance [46].

Here, we identify deletions of several genes (*bug1Δ, bem1Δ, arf1Δ, hog1Δ, las17Δ, vps5Δ,* and *sac6Δ*) that reduce the efficiency with which overexpression of Sup35PD can induce the *de novo* appearance of [*PSI* ^+^]. Deletion of *LAS17, VPS5,* or *SAC6*, which are associated with endocytosis and the actin cytoskeleton, not only inhibit [*PSI* ^+^] induction, but also suppress the toxicity and aggregation associated with the expanded glutamine repeats of the huntingtin protein exon 1 that causes Huntington's disease.

## Results

### Deletions of *BUG1*, *BEM1*, *ARF1*, *HOG1*, *LAS17*, *VPS5*, and *SAC6* show low or no induction of [*PSI* ^+^] but maintain propagation of [*PSI* ^+^] and [*PIN* ^+^]

Our goal was to identify genes that influence the induction of the [*PSI^+^*] prion. Previous work approached this problem by making use of the observation that overexpression of Sup35PD-GFP in [*PSI^+^*] [*PIN* ^+^] cells causes toxicity due to excessive sequestration of essential Sup35 into large aggregates [46], [47]. When Sup35PD-GFP is highly overexpressed in [*psi^−^*] [*PIN* ^+^] cells, the frequent induction of [*PSI* ^+^] results in an intermediate level of toxicity, because only cells that have switched to the [*PSI* ^+^] state are sick [23], [46]. Therefore, genes whose deletions enhance or reduce the toxicity associated with overexpression seemed likely to increase or decrease [*PSI* ^+^] induction frequency, respectively [46]. We tested 238 deletion strains that enhanced toxicity, 151 that reduced toxicity, and nine other strains studied in Tyedmers *et al*. [46] (Table S1; Tyedmers and Lindquist, unpublished) for their effects on [*PSI* ^+^] induction.

First, to distinguish the inability to induce [*PSI* ^+^] from the inability to propagate [*PSI* ^+^] we tested if the 398 deletions could maintain weak and strong variants of [*PSI* ^+^] in a propagating culture after cytoduction with [*PSI* ^+^]. A plasmid encoding a copper inducible Sup35PD-GFP fusion was plasmiduced into the deletion strains simultaneously with the cytoduction of weak or strong [*PSI* ^+^]. After over 50 generations of growth, we scored for maintenance of [*PSI* ^+^] by overexpressing Sup35PD-GFP and examining cells for the presence of fluorescent aggregates. All deletion strains cytoduced with either weak or strong [*PSI* ^+^] contained fluorescent aggregates, indicative of [*PSI* ^+^], except for *hsp104Δ,* which had the characteristic diffuse fluorescence of [*psi^−^*] cells (data not shown). Additionally, we have previously shown that [*PIN* ^+^] is maintained in all strains of the deletion library except *rnq1Δ* and *hsp104Δ* [48].

Next, we used a standard nonsense suppression assay for [*PSI* ^+^] to screen the 398 yeast deletion library strains, in the BY4741 background, for their effects on induction of stable propagating [*PSI* ^+^]. To do this, the [*PIN* ^+^] prion, the Sup35PD-GFP plasmid, and a plasmid containing a *ura3*–*14* nonsense allele to score for [*PSI* ^+^] cells [49] were simultaneously cytoduced into the 398 deletion strains as described previously [48]. Following overexpression of Sup35PD-GFP to induce [*PSI* ^+^], cells were plated on–Ura where suppression of the plasmid borne *ura3*–*14* nonsense allele allowed [*PSI* ^+^], but not [*psi^−^*], cells to grow.

**Figure 1:**
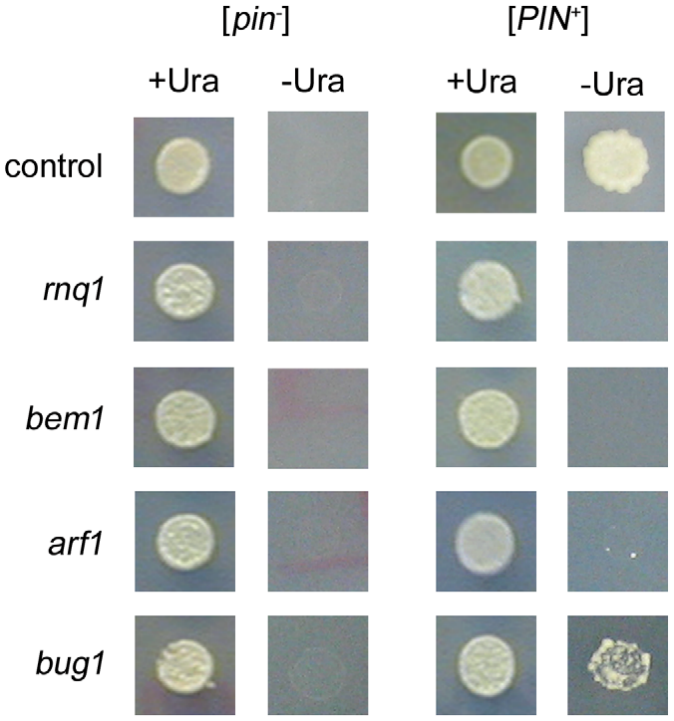
Scoring for [*PSI* ^+^] induction in deletion strains. All deletion library candidates in the BY4741 background were cured on GuHCl and cytoduced using *kar1* donor strains containing the *Sup35PD-GFP* and *ura3*–*14* plasmids and either high [*PIN* ^+^] (right) or [*pin* ^−^] (left). Expression of *Sup35PD-GFP* was induced on plasmid selective media containing 50 µM copper for two days and then spotted either onto -Ura to score for [*PSI* ^+^] induction or +Ura to assay growth. All cytoductions were performed in duplicate and cytoductants were tested for the ability to induce [*PSI* ^+^] multiple times. Control strains (top row) show growth on -Ura in [*PIN^+^*], but not [*pin* ^−^], strains after 8 days. Both *rnq1Δ* (second row) and *bem1Δ* (third row) strains show no growth on -Ura after 11 days, indicative of no [*PSI* ^+^] induction. Deletion strains showing very low induction, e.g. *arf1Δ*, exhibit very few colonies after 11 days of growth on -Ura (fourth row), whereas strains showing low induction (e.g. *bug1Δ*, last row) show slow growth on -Ura after eight days.

Six novel deletion strains: *bem1Δ, def1Δ, scp160Δ, rpp1aΔ, spt4Δ, and pre9Δ,* as well as the expected *rnq1Δ* and *hsp104Δ* deletions, failed to grow on -Ura (Table 1). Previous work has shown that in the presence of the *SUP35 R2E2* allele, which increases the appearance of [*PSI* ^+^] without *SUP35* overexpression, *bem1Δ* and *pre9Δ* decreased [*PSI* ^+^] appearance [46]. In addition, 29 other deletions showed either low or extremely low induction of [*PSI* ^+^] (Figure 1; Table 1). While strains carrying deletions of *SPT4* or *YML010C-B* failed to express Sup35PD-GFP, all other deletion strains expressed Sup35PD-GFP at similar levels (data not shown).

**Table 1:** Yeast deletion library strains that show no, very low, or low induction of [*PSI* +].

Deletions that fail to induce [*PSI* +]			
*bem1Δ* * ^-^	*hsp104Δ* -	*rnq1Δ* * *^-^*	*scp160Δ* * ^-^
*def1Δ* * ^-^	*pre9Δ* *	*rpp1aΔ* * ^+^	*spt4Δ* ‡ ^-^

Deletions that show very low induction of [*PSI* +]			
*ase1Δ* * *^-^*	*bfr1Δ* * *^-^*	*hog1Δ* * *^+^*	*rpl20bΔ* * *^-^*
*arf1Δ* * *^-^*	*bre1Δ* * *^+^*	*hop2Δ* † ^-^	*ycl005wΔ* † ^-^

Deletions that show low induction of [*PSI* +]			
*ada*2*Δ* +	*nas2Δ* * *^+^*	*snf8Δ* +	*ydr048cΔ* † ^+^
*asc1Δ* +	*new1Δ* +	*swa2Δ* * *^+^*	*yhr033wΔ* +
*bug1Δ* * *^+^*	*pub1Δ* -	*trf5Δ* * *^+^*	*yml010c-bΔ* ‡ ^-^
*cik1Δ* * *^+^*	*sac1Δ* +	*tsr2Δ* +	
*gvp36Δ* +	*sgf73Δ* +	*vid22Δ* +	
*lst7Δ* * ^+^	*slg1Δ* +	*ydl032w* +	

*Re-engineered deletion.

**†:** Unable to obtain deletion.

**‡:** Showed extremely low induction of Sup35PD-GFP.

**+:** enhanced toxicity as described by Tyedmers *et al*., unpublished.

- reduced toxicity as described by Tyedmers *et al*., unpublished.

Deletion strains that decrease the efficiency of [*PSI* ^+^] induction (Table 1) were from both enhanced and reduced toxicity groups. This makes sense if we consider that toxic side effects of the deletions could overlay the positive effect on growth rate due to the reduced [*PSI* ^+^] induction. Furthermore, in some deletions, even the overexpression of YFP alone (without the Sup35 prion domain) causes strong toxicity that must be, therefore, completely unrelated to prion induction frequency [46].

**Figure 2:**
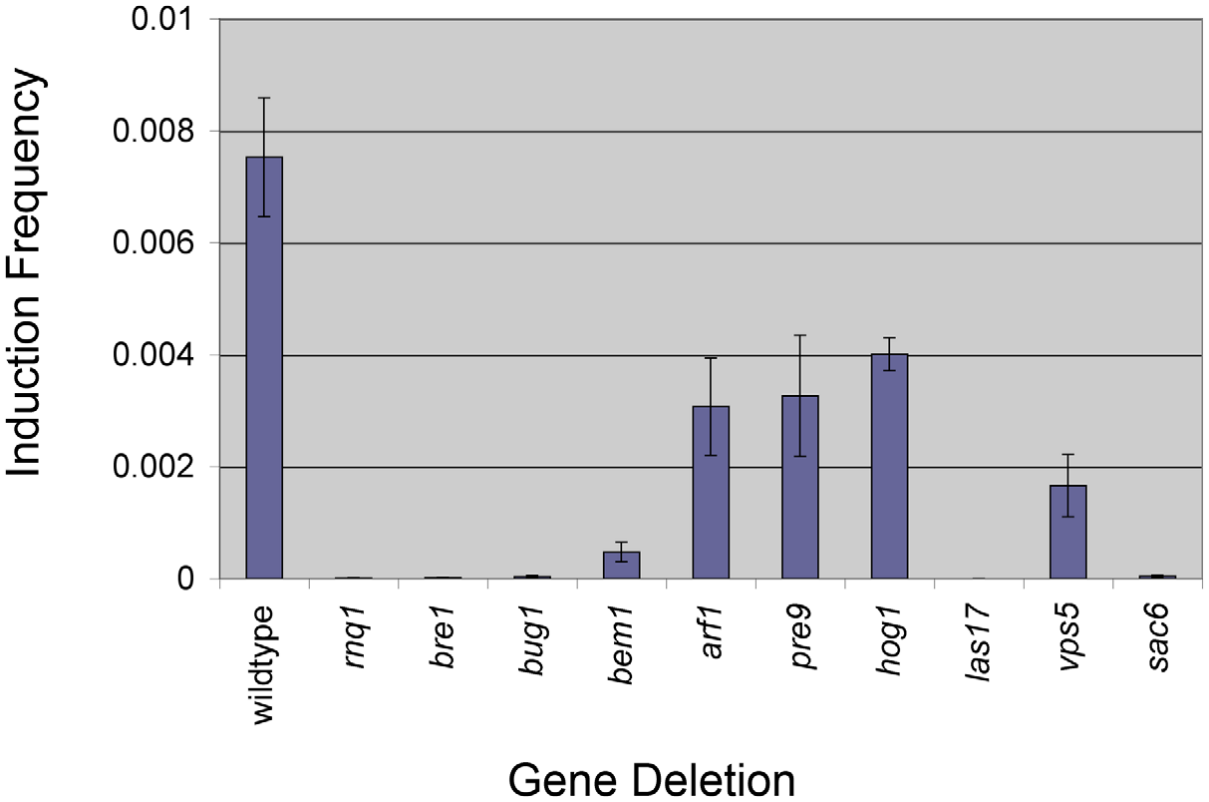
Nine deletions show reduced [*PSI* ^+^] appearance. Genes were disrupted in the 74D-694 [*PIN* ^+^] strain background. Deletion strains, transformed with the *Sup35PD-GFP* plasmid, were grown in 10 ml of plasmid selective media supplemented with copper for 24 hours at 30°C and plated on SD-Ade in order to determine [*PSI^+^*] induction frequency. Induction frequency and standard error were calculated with at least one transformant from each independent knock out (see Table S3) for a total of three transformants per deletion. The exception was *pre9Δ* which was calculated from three transformants from a single independent knock out. Only deletion strains that showed a significant difference in induction frequency compared to wildtype strains (*p*<0.05 unpaired t-test) were included in the figure.

To eliminate the effects of secondary mutations known to have accumulated in library strains [50], [51], multiple independent deletions of nineteen of the best candidates that showed no or reduced induction of [*PSI* ^+^] (Table 1) were re-engineered in a wildtype 74-D694 [*PIN* ^+^] strain. This 74-D694 genetic background contains a [*PSI* ^+^] suppressible *ade1*–*14* allele that provides the ability to directly score for [*PSI* ^+^] by examining growth on -Ade. Of the 19 re-engineered deletions, only the six deletions (*bre1Δ, bug1Δ, bem1Δ, arf1Δ, pre9Δ,* and *hog1Δ*) that reproducibly reduced the frequency of [*PSI* ^+^] induction, relative to the wildtype induction frequency of approximately 7.5 X 10^−3^ (Figure 2), were pursued further. Since a slow growth phenotype complicates the scoring for [*PSI* ^+^], deletions that significantly inhibited growth in the 74D-694 background, like *lst7Δ* and *swa2Δ* (data not shown), were eliminated from further analysis.

In addition to the above deletions, we made a deletion of *LAS17,* which was previously shown to inhibit the aggregation of the polyglutamine 103Q repeat [52]. We also made deletions of *VPS5* and *SAC6,* because they, like Las17 and other proteins shown to affect the appearance of [*PSI* ^+^], are associated with endocytosis and the actin cytoskeleton. Even though prion propagation is unaffected in these three deletion strains because they were all able to maintain [*PSI* ^+^] over many generations after cytoduction (data not shown), *vps5*Δ caused a significant decrease in prion induction, and *las17Δ* and *sac6Δ* strains completely failed to induce [*PSI* ^+^] (Figure 2).

To eliminate deletions that reduced [*PSI* ^+^] induction by altering the levels of Sup35, Sup35PD-GFP, or chaperones, we examined these levels in all strains. None of the deletions caused significant changes in Hsp104, Ssa1, Sis1, Sse1, or Ssb1/2 (data not shown). Since Sup35PD-GFP levels were reduced in *bre1Δ* strains and Sup35 endogenous levels were decreased in *pre9Δ* strains (Figure S1), these deletions were dropped from further study.

To eliminate the possibility that the reduced [*PSI* ^+^] induction was due to the loss of [*PIN* ^+^] during the construction of any of the deletion strains, we showed that the maintenance of [*PIN* ^+^] was unaffected by the deletions. Each independently constructed deletion was crossed to a [*pin* ^-^] strain carrying a plasmid with the *CUP1* controlled *RNQ1-GFP* fusion. When grown on medium containing copper, induction of the resulting diploid containing the *RNQ-GFP* construct caused the appearance of punctate dots indicative of [*PIN* ^+^] (data not shown). Furthermore, we showed that [*PIN* ^+^] was maintained in deletion strains by the presence of [*PIN* ^+^] characteristic SDS-resistant oligomeric species after 24 hours of Sup35PD-GFP overexpression (Figure S2). Differences in the migration of Rnq1 SDS-resistant oligomers have been shown to be associated with different [*PIN* ^+^] variants [53]. We observed similar migration of Rnq1 oligomeric species in the deletion strains, suggesting that the [*PIN* ^+^] prion variants were not altered during construction of the strain or by Sup35PD-GFP overexpression. These results and other properties of the deletions investigated further below are summarized in Table 2.

**Table 2:** Properties of deletions that inhibit *de novo* induction of [*PSI* ^+^].

Deletion	Toxicity with Sup35PD over-expression [46]	Reaches stationary phase after 24 hours	Maintains [*PIN* ^+^]	Maintains [*PSI* ^+^]	[*PSI* ^+^] induction	Detection of Sup35PD-GFP rings	Viability of ring cells	103Q bright aggre-gates	Growth of [*PIN* ^+^] 103Q xpressing cells	Function of gene	Mammalian homolog
Wildtype	N/A	+	+	+	+	+	+ (35% ±4.4)	+	+/-	N/A	N/A
*bug1Δ*	enhanced	+	+	+	- - -	+	++ (59% ±6.1)	-a	- -	Involved in vesicle trafficking in anterograde ER to Golgi transport	GM130 [74]
*bem1Δ*	reduced	+	+	+	-	+	+ (47% ±6.0)	-a	- -	Activates Cdc42 to polymerize actin for bud formation [75], [76]	None
*arf1Δ*	reduced	+	+	+	-	+	+/- (22% ±4.2)	-a	-	Initiates actin assembly on golgi membranes to drive retrograde transport [77]	Arf1 [78]
*hog1Δ*	enhanced	+	+	+	- - -	+	+ (44% ±2.5)	-a	-	Associated with osmoregulation and possibly involved in actin polymerization in the presence of low pH [79]	MAPK p38 [80]
*las17Δ*	unknown	+/-	+	+	- -	- -	unknown	- - -b	+	Actin assembly factor at endocytic sites	Wiscott-Aldrich syndrome protein [81]
*vps5Δ*	unknown	+	+	+	- - -	-	- (13% ±2.6)	- - -	+	Actin bundling protein at endocytic sites	SNX1 [82]
*sac6Δ*	no effect	+	+	+	- - -	+/-	unknown	- - -	+	Vesicle trafficking in endosome to golgi	Fimbrin [83]

**Figure 3:**
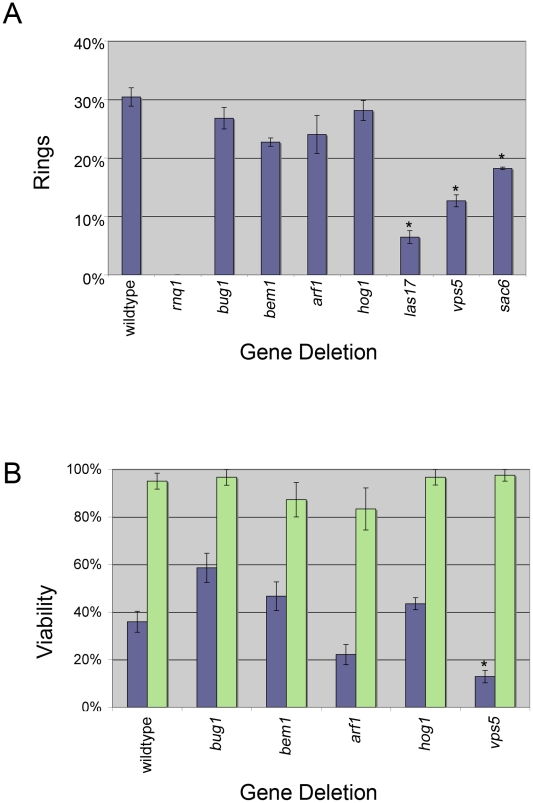
Ring formation and viability. A. [*PIN* ^+^] cells containing *Sup35PD-GFP* were induced in copper containing liquid media for 24 hours. The percentage of cells containing rings was determined by counting more than 300 cells from at least one transformant from each independent knockout line (see Table S3) for a total of three transformants per deletion. Bars indicate standard error and (*) indicates deletion strains that showed a significant difference in the percentage of cells containing rings compared to wildtype (*p*<0.01 unpaired t-test). B. Ring containing cells (purple bars) or cells with diffuse cytoplasmic fluorescence (green bars) were isolated by micromanipulation, placed on rich media and assayed for growth. At least 20 ring containing cells and 10 diffuse cells, from at least one transformant from each independent knock out line for a total of three transformants, were tested. Bars indicate standard error and (*) indicates the deletion strains that showed a significant difference in viability compared to wildtype strains (*p*<0.01 unpaired t-test).

**Figure 4:**
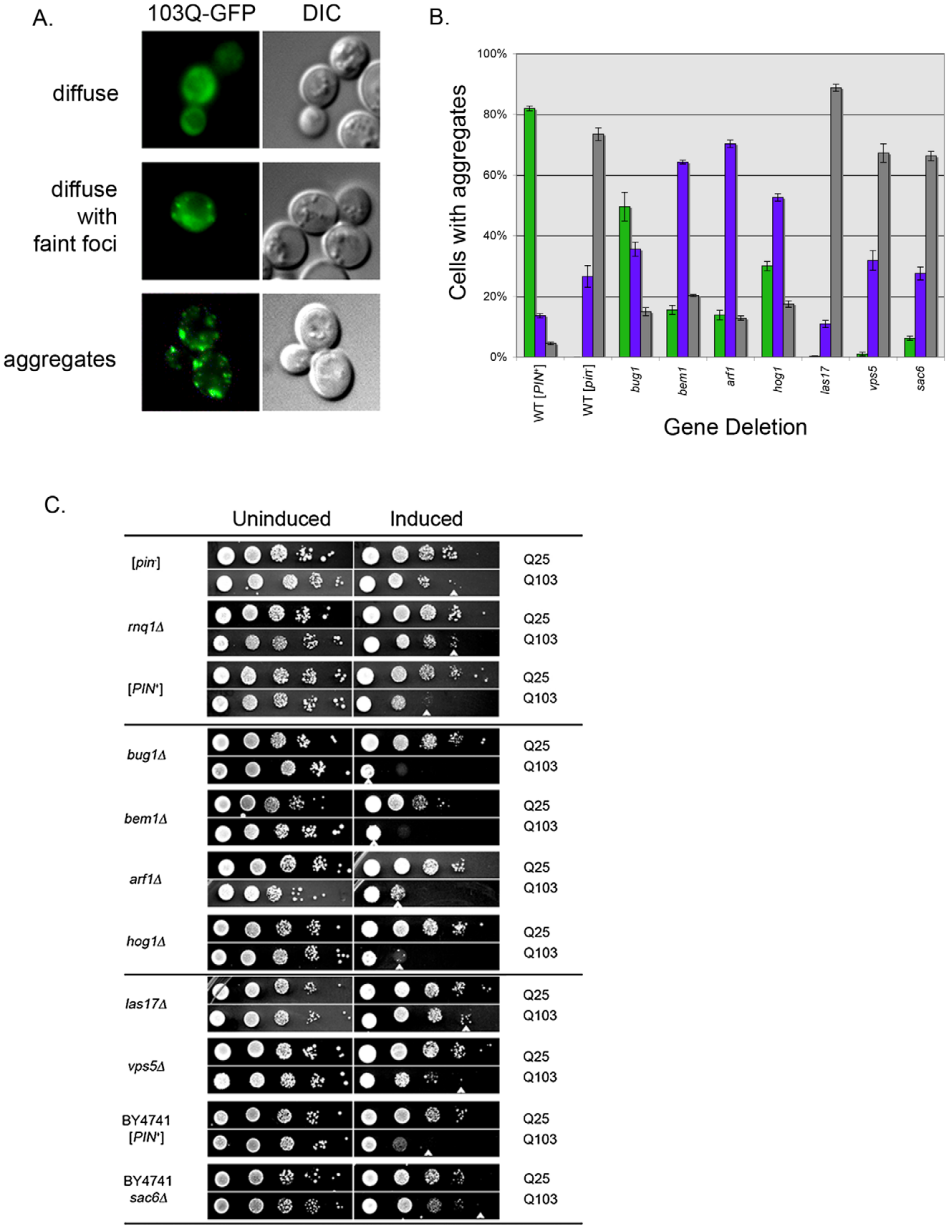
Aggregation and toxicity of 103Q in deletion strains. A. Log phase wildtype [*PIN* ^+^] cells containing the 103Q-GFP plasmid were induced in galactose containing media for one to two hours and then visualized. Within the population, most cells exhibited strong fluorescent aggregates on a non-fluorescent background (bottom), some cells had faint fluorescent foci on a cloudy fluorescent background (middle), and a very small population of cells had diffuse cytoplasmic fluorescence (top). B. Deletions containing 103Q-GFP were induced and scored for the different cell types indicated in (A). The percentages of cells containing strong fluorescent aggregates (green bars), faint foci (purple bars), and diffuse cytoplasmic fluorescence (gray bars) were calculated from over 300 cells from at least one transformant from each independent knock out line for a total of three transformants. C. Wildtype [*PIN* ^+^], wildtype [*pin* ^−^], *rnq1Δ,* and the indicated [*PIN* ^+^] deletion strains were transformed with either the 103Q-GFP or 25Q-GFP plasmid and grown to late log phase in glucose (uninducing) media. Cells were serially diluted 20-fold and plated on media that either induced (right column; galactose containing media) or did not induce (left column; glucose containing media) the overexpression of the 25Q-GFP or 103Q-GFP plasmid. White arrowhead indicates the lowest dilution displaying growth. Deletion strains in (C) are in the 74D-694 background except *sac6Δ*, which is in the BY4741 [*PIN* ^+^] background as indicated. Pictures shown are representative of the multiple independent deletion lines tested for 103Q-GFP toxicity.

All strains contain the [*PIN* ^+^] prion. Deletion strains were assessed compared to wildtype. (-) indicates a lower degree compared to wildtype. Whether strains exhibited enhanced or reduced toxicity during Sup35PD overexpression in Tyedmers *et al*. [46] is listed under ‘Toxicity with Sup35PD overexpression’. ‘Reaches stationary phase after 24 hours’ indicates which deletion strains containing *Sup35PD-GFP*, when induced with copper for 24 hours, reach stationary phase (Figure S3). Strains carrying a deletion of *LAS17* took 36 hours to reach stationary phase. ‘Maintains [*PIN* ^+^]’ and ‘Maintains [*PSI* ^+^]’ indicate strains that can propagate the respective prion after cytoduction. ‘[*PSI* ^+^] induction’ indicates the relative induced frequency of [*PSI* ^+^] formation (Figure 2). ‘Detection of Sup35PD-GFP rings’ indicates the fraction of cells containing rings after 24 hours of *Sup35PD-GFP* overexpression (Figure 3A), whereas the ‘viability of ring cells’ refers to toxicity associated with ring containing cells (Figure 3B). The frequency of cells with bright 103Q-GFP aggregates accumulating after one to two hours of induction (Figure 4B) are summarized under “103Q bright aggregates.” The effect of 103Q-GFP overexpression on the growth of deletions strains are under ‘Growth of [*PIN* ^+^] 103Q-GFP expressing cells’.

a aggregates were reduced, but faint aggregates on diffuse background were increased.

b orted previously by [52].

### Reduced [*PSI* ^+^] formation is not always associated with reduced ring formation

Transient overexpression of Sup35PD-GFP in [*psi^−^*] [*PIN* ^+^] cells leads to the appearance of cytoplasmic fluorescent rings and lines [54]. Daughter cells derived from micromanipulated cells that contain such rings or lines, where overexpression of Sup35PD-GFP is turned off but where endogenous *SUP35* is tagged with GFP, always contained fluorescent punta indicative of [*PSI* ^+^] [55]. In contrast, mother cells without ring or line aggregates always gave rise to daughter cells with diffuse fluorescence, indicative of [*psi^−^*] [44], [54]–[56]. Thus the appearance of rings/lines, while not necessarily a direct intermediate in the formation of [*PSI* ^+^], is nonetheless a hallmark of the potential appearance of induced [*PSI* ^+^]. No such rings have ever been observed during spontaneous appearance of [*PSI* ^+^] in the absence of Sup35PD overexpression (unpublished).

To determine the deletions that inhibit the induction of [*PSI* ^+^] and also prevent ring formation, we compared the number of cells that contained rings after 24 hours of expressing Sup35PD-GFP in the seven deletion strains. Ring containing cells in four of the deletions, *bug1Δ, bem1Δ, arf1Δ,* and *hog1Δ*, appeared at levels similar to wildtype (30%, Figure 3A). The *rnq1Δ* strains, which are [*pin* ^−^], always displayed diffuse fluorescence (Figure 3A), as expected, since [*PIN* ^+^] has been previously shown to be required for ring formation [54].

Interestingly, when measured after 24 hrs of Sup35PD-GFP induction, the three deletions associated with endocytosis and organization of the actin cytoskeleton, *sac6Δ, las17Δ,* and *vps5Δ*, all showed a significant reduction in cells containing rings. Since by 25 hrs of induction, wildtype, *sac6Δ,* and *vps5Δ* cultures were in stationary phase, where ring formation peaks [54], the inhibition of ring formation in *sac6Δ and vps5Δ* was not the result of a failure of these cultures to reach stationary phase (Figure S3). Also, since *las17Δ* cells reached saturation phase only after 36 hours, we also measured ring formation in *las17Δ* cultures following 50 hrs of induction, well after the culture reached stationary phase. These cultures still showed a 50% reduction in ring formation relative to the wildtype cultures (data not shown).

### Ring-associated toxicity

Ring formation is associated with [*PSI* ^+^] appearance [54]. However, it has been shown that cells that contain rings have a higher rate of cell death than those that have diffuse fluorescence [44], [47], [54]. Therefore, we asked if the deletions might be more toxic to ring bearing cells, which would result in a decrease in [*PSI* ^+^] induction. To test this, we micromanipulated individual ring containing cells from selected deletion strains and assessed whether they were viable. Similar to previous findings [47], only 36% of wildtype ring containing cells were viable, whereas 95% of wildtype cells with diffuse cytoplasmic fluorescence were viable (Figure 3B). In general, the viability of ring containing wildtype, *bem1*Δ, and *hog1*Δ cells appeared to be similar (Figure 3B) and therefore cannot explain the strong reduction of *de novo* induction of [*PSI* ^+^]. Ring containing *arf1Δ* cells had reduced viability (21%), which could account for the decrease in [*PSI* ^+^] induction (Figure 2). In contrast, *bug1Δ* ring cells showed an increase in viability (59%), but apparently these cells did not efficiently give rise to [*PSI* ^+^] cells. In the absence of rings, there appeared to be no effect on the viability of any of the deletion strains (Figure 3B, green bars).

Since isolating ring cells in strains with low ring formation was difficult, we focused on one example and found that of the small population of cells in *vps5Δ* strains that formed rings, a majority were inviable (Figure 3B). This suggests that rings in the absence of *VPS5* may be harmful to the cell and likely explains the decreased percentage of rings observed in Figure 3A.

### Deletion strains affect aggregation and toxicity of polyglutamine

We next asked if our deletion strains also affect the [*PIN^+^*] dependent aggregation of the polyglutamine (polyQ) expanded repeat found in the mutant huntingtin protein associated with Huntington's disease. Since *las17Δ* strains, carrying a galactose inducible expanded polyQ repeat (103Q) fused to GFP, were previously shown to delay 103Q-GFP aggregate formation compared to wildtype strains [52], we tested our other deletions for similar aggregation patterns. When wildtype [*PIN^+^*] cells expressed 103Q-GFP for approximately one to two hours, 82% of cells displayed strong fluorescent puncta, while [*pin^-^*] cells showed mostly cells with diffuse fluorescence (73%) and a minor population that contained a few faint fluorescent foci on a diffuse background (27%; Figure 4A and 4B). Similar analyses of [*PIN* ^+^] strains with *bug1Δ, bem1Δ, arf1Δ,* and *hog1Δ* deletions resulted in a reduced number of cells that contained strong fluorescent puncta (Figure 4B, green bars) and an increased number of cells that had faint fluorescent foci with a diffuse background (Figure 4B, purple bars). As in the previous study [52], [*PIN* ^+^] *las17Δ* strains had barely any strong fluorescent foci. Examination of [*PIN* ^+^] *vps5Δ* and [*PIN* ^+^] *sac6Δ* strains also showed a strong reduction in cells having bright foci and a similar distribution of cells with faint foci vs. no foci as seen in wildtype [*pin* ^-^] strains.

We next examined the effect of these deletions on toxicity associated with expression of the expanded polyglutamine protein in the presence of the [*PIN* ^+^] prion [57]. Our controls verified earlier findings [57] that wildtype [*PIN* ^+^] cells carrying a galactose inducible 103Q-GFP plasmid show toxicity compared to cells carrying the non-toxic 25Q-GFP plasmid. As expected, polyglutamine expressing cells that are either [*pin* ^-^] or *rnq1Δ* did not exhibit toxicity, whereas wildtype [*PIN* ^+^] cells were sick (Figure 4C). Since previous studies have correlated the presence of polyQ aggregates with cell toxicity [57], the lack of strong polyQ fluorescent foci in the class of deletions that also reduced ring formation after Sup35PD-GFP overexpression (*las17Δ, vps5Δ,* and *sac6Δ*; Figure 4A and B) nicely explained the observed decrease in polyQ toxicity. Interestingly, the class of deletions that reduced [*PSI* ^+^] induction, but not ring formation (*bug1Δ, bem1Δ, arf1Δ,* and *hog1Δ),* increased the frequency of cells containing faint polyQ-GFP fluorescent foci on diffuse backgrounds and clearly enhanced 103Q toxicity.

## Discussion

We scored 398 gene deletions, previously identified by their ability to increase or decrease toxicity caused by overexpression of Sup35PD-GFP [46], for effects on the induction of [*PSI* ^+^] and established effects for four of the deletions (see Table 2 summarizing all results). Since there is not always a strong correlation between the toxicity associated with *SUP35* overexpression and the induction of [*PSI* ^+^], we tested all of the enhanced and reduced toxicity candidates found in the previous study [46] for [*PSI* ^+^] induction. We observed that induction of [*PSI* ^+^] was inhibited by deletions that either reduced (*arf1Δ* and *bem1Δ*) or enhanced (*bug1Δ* and *hog1Δ*) toxicity. Possibly the rings or other less visible aggregates have an altered property in the presence of *bug1Δ* or *hog1Δ* that inhibits detectable [*PSI* ^+^] appearance and causes toxicity. It has been shown that rings cause toxicity by titrating Sup35 and/or Sup45 (the essential release factor that binds to Sup35) into aggregates and away from the ribosome [46]. The toxicity associated with *bug1Δ* and *hog1Δ* during [*PSI* ^+^] induction could be an enhancement of this effect or could be by the formation of a different type of toxic [*PSI* ^+^] intermediate.

We also examined three deletions (*las17Δ*, *vps5Δ,* and *sac6Δ*), which were chosen for their association with endocytosis and the actin cytoskeleton, and found that they reduce [*PSI* ^+^] induction. While *las17Δ* and *vps5Δ* were not included in the library originally scored for SUP35PD-GFP toxicity, the *sac6Δ* BY4741 library strain was not associated with changes in toxicity [46]. We found that *sac6Δ* inhibits [*PSI* ^+^] induction in both the deletion library (BY4741; data not shown) and the 74-D694 (Figure 2) backgrounds. This suggests that other non-essential deletions that inhibit [*PSI* ^+^] induction were likely missed in the toxicity screen.

All seven of our deletion strains reduce [*PSI* ^+^] induction caused by overexpression of Sup35PD-GFP. While a deletion of *BEM1* was previously shown to reduce the spontaneous appearance of [*PSI* ^+^] associated with the *SUP35* R2E2 expanded repeat [46], it is unknown whether our deletions affect the spontaneous appearance of [*PSI* ^+^] without overexpression or mutated alleles (e.g. R2E2) of Sup35. Since the spontaneous appearance of [*PSI* ^+^] is very infrequent and Mendelian suppressors with the phenotype of [*PSI* ^+^] [58] appear at a higher rate than [*PSI* ^+^], scoring for mutations that lower the appearance of [*PSI* ^+^] is challenging.

### Prion and polyglutamine formation and maturation

**Figure 5:**
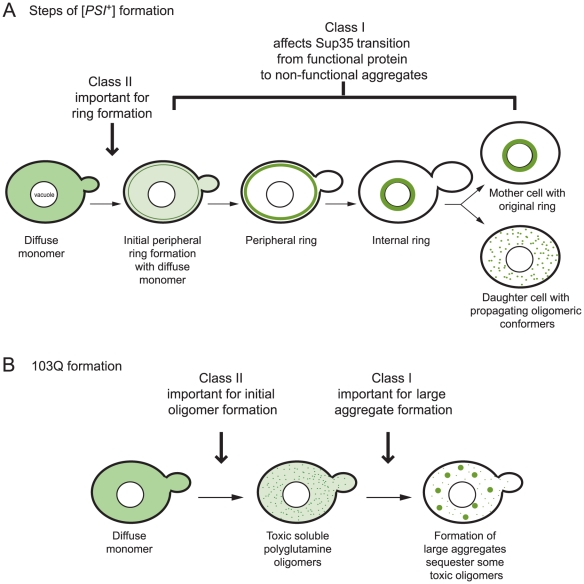
Proposed effects of class I and II genes on the multistep pathways of *de novo* prion and polyglutamine aggregate formation. A. Induction of [*PSI^+^*] by transient overexpression of *Sup35PD-GFP* in a [*psi^−^*] [*PIN^+^*] strain with endogenous Sup35 tagged with GFP. As described in Mathur *et al*., [55] the first stages of [*PSI* ^+^] induction involve the formation of a peripheral fluorescent ring. Class II deletions appear to inhibit this step. As the ring matures, diffuse Sup35 diminishes in intensity. The peripheral ring then collapses to surround the vacuole. The daughters of the ring containing cell contain multiple Sup35:GFP aggregates characteristic of [*PSI^+^*]. Class I deletions interfere with transitioning to [*PSI* ^+^] after the ring appears. B. Hypothetical model of 103Q aggregation in [*PIN* ^+^] cells. Class II proteins function in the initial formation of small toxic 103Q oligomers (left), since class II gene deletions result in diffuse fluorescence and suppression of toxicity. Class I proteins most likely facilitate incorporation of these small oligomers into large protective aggregates (right).

Time lapse examination of individually micromanipulated [*psi^−^*] [*PIN* ^+^] cells, containing an endogenously tagged *Sup35-GFP* fusion and transiently overexpressing *Sup35PD-GFP* from a plasmid, previously showed that a fluorescent ring initially forms at the cell periphery and then internalizes around the vacuole. Later, such cells with rings give rise to [*PSI* ^+^] daughter cells with fluorescent foci (Figure 5A) [44], [55].

In this paper, we identified two classes of gene deletions that reduce the *de novo* induction of stable [*PSI^+^*] but differ in effects on ring formation. Class I deletions (*bug1Δ, bem1Δ, arf1Δ,* and *hog1Δ)* form Sup35PD-GFP rings at approximately wildtype levels (Figure 3). Class II deletions (*las17Δ*, *vps5Δ,* and *sac6Δ*) have a significantly reduced number of cells with Sup35PD-GFP rings (Figure 3).

The existence of these two deletion classes suggests that the prion formation pathway can be inhibited at different steps. In the case of class I deletions, problems in peripheral and internal ring formation were not detected (data not shown), suggesting that these genes are important for ring containing cells to transmit heritable [*PSI* ^+^] aggregates to daughter cells (Figure 5A). In the case of class II deletions, peripheral rings form infrequently, suggesting that these genes are important in the initial formation of the ring even in the presence of [*PIN* ^+^] (Figure 5A). While rings contained in *vps5*Δ (class II) cells have reduced viability, it is unlikely that ring formation is so toxic that cells die before the ring appears because there is no corresponding increase in toxicity in non-ring containing cells (Figure 3B).

The formation of polyglutamine aggregates, upon overexpression of Q103:GFP in a [*PIN^+^*] strain, is not associated with ring formation. Instead, large bright aggregates are observed within one to two hours of induction [52]. All of our deletions affected polyglutamine aggregation, but the type of effect differed based on the class of the deletion. Class I deletions (*bem1Δ*, *bug1Δ*, *arf1Δ,* and *hog1Δ*) caused a decreased level of bright fluorescent aggregates and an increase in diffuse cells with faint foci (Figure 4A and 4B). Interestingly, these deletions enhanced the toxicity associated with 103Q-GFP (Figure 4C). In the presence of the class II deletions (*las17Δ, vps5,* and *sac6Δ*) 103Q-GFP usually remained diffuse, and very few cells had bright aggregates (Figure 4B). Additionally, all class II deletions suppressed polyglutamine toxicity (Figure 4C).

The formation of large huntingtin aggregates in mammals was initially thought to be the cause of huntingtin-mediated cell death (reviewed in [59]), but emerging evidence suggests that these large aggregates are neuroprotective ([60], reviewed in [61]) and toxicity is due to a soluble pool of oligomeric conformers [62]-[63]. Also in yeast, small multiple foci of 103Q appear to be more toxic than a large aggregate [64], [65]. Thus, we propose that class I genes are important for the formation of large protective aggregates but not small toxic oligomers (Figure 4B), and that when a class I gene product is absent, the reduction of large protective aggregates permits the increased propagation of deadly soluble oligomers.

In contrast, in cells with class II deletions, 103Q-GFP toxic oligomers or aggregates may form only rarely. While the [*PSI* ^+^] and polyglutamine formation pathways are not directly comparable, we propose that class II genes affect the initial steps of polyglutamine formation, and the formation of large protective aggregates (promoted by class I genes) is a downstream event (Figure 5B).

### Actin and prion formation

Although endocytosis is relatively unaffected in *bem1Δ, bug1Δ, arf1Δ, hog1Δ, las17Δ, vps5,* and *sac6Δ* strains, six out of seven of our deletion mutants display fragmented vacuoles (Figure S4) [66], [67]. Possibly, vacuole fragmentation may affect the perivacuolar deposition site for aggregated proteins, called IPOD [68], which has been suggested to play a role in prion formation [55], [56]. Some of the deletion library strains we screened induced [*PSI* ^+^] normally but had fragmented vacuoles (Table SI, asterisk; [66]), suggesting that intact vacuoles are not necessarily a requirement for efficient prion appearance. Vacuole fragmentation has been shown to be associated with microtubular defects [69] as well as fluctuations in the soluble actin pool [67]. We showed that treatment with the microtubule disrupting drugs Nocodazole (Figure S4B and S4C) and Thiabendazole (data not shown) at concentrations that did not inhibit Sup35PD-GFP induction do not affect [*PSI* ^+^] appearance. Conversely, the actin disrupting drug Latrunculin A [70] and *act1-R117A* alleles [44] do inhibit [*PSI* ^+^] induction. Furthermore, many of the deletions identified in this study are involved with actin (Table 2), suggesting that actin organization plays a critical role in the aggregation of prion and polyglutamine proteins.

Possibly, proper actin organization on the cell periphery is required for the initial formation of the [*PSI^+^*] ring or polyglutamine oligomers, where as actin organization elsewhere in the cell could be required for downstream events such as the formation of a propagating [*PSI* ^+^] conformer or the formation of large protective polyglutamine aggregates. Actin could possibly be involved in facilitating the addition of monomer to newly formed aggregates. In assaying for [*PSI* ^+^], ring cells containing functional monomer not yet integrated into an aggregate would appear to be [*psi^−^*].

Our data suggests that prion and polyglutamine formation involves a multi-step process that is dependent upon actin organization. Interestingly, six of the seven proteins identified here have mammalian homologues (Table 2), suggesting that similar mechanisms may be involved in aggregation and oligomerization of QN-rich proteins in higher eukaryotes. Further elucidation of how actin nucleation contributes to prion induction will not only shed light on how toxic oligomeric species are formed, but also could provide clues to the molecular mechanisms underlying many human aggregating neurodegenerative diseases.

## Methods

### Plasmids and strains

In this work, 398 yeast deletion library strains (parent strain BY4741: *MAT* ***a*** *ura3Δ his3Δ1 leu2Δ met15Δ*; Open Biosystems, Huntsville, AL; Table S1) previously obtained from an earlier toxicity screen ([46]; Tyedmers and Lindquist unpublished) were scored for effects on [*PSI* ^+^] induction in a wildtype Sup35 background. The *“kar1* plasmid donor” strain (GF667; *MATα CEN1–16::pGal1-CEN1–16-URA3^Kl^ kar1Δ15 lys2 rad5-535 leu2-3,112 can1-100 his3-11,15 trp1-1 cyh^R^*) was used to introduce plasmids and prions into the deletion library strains via cytoduction, as described in Manogaran *et al*. [48]. A plasmid containing a copper inducible prion and middle domain of Sup35 (Sup35PD) fused to green fluorescent protein (Sup35PD-GFP; p1181: *CEN2 HIS3 ori ARS Amp^R^ pCup1-Sup35PD-GFP*) was used to induce [*PSI* ^+^] in deletion strain derivates of BY4741, while a *LEU2* version of the plasmid (p1182) was used in 74-D694 (L1749; [33]; *MAT* ***a*** *ade1*–*14 leu2*–*3,115 his3Δ200 ura3*–*52 trp1*–*289* high [*PIN* ^+^]). To score for [*PSI* ^+^] in the BY4741 strains, a plasmid containing the [*PSI* ^+^] suppressible *ura3*–*14* allele ([49]; p1513; *CEN2 LEU2 ura3*–*14 ori ARS Amp^R^*) was used. A tester strain (L2174; *MATα leu2 ura2 his3* [*pin* ^-^]) transformed with a copper inducible *RNQ1* fused to GFP (p1186; *CEN LEU2 ori ARS AmpR pCUP1-RNQ1:GFP)* was used to confirm the presence of [*PIN^+^*] in 74D-694 deletion strains (see below). Plasmids p1572 and p1838 [52], which contain a fusion of GFP to the galactose inducible 25Q or 103Q repeats in exon 1 of the huntingtin gene, respectively, were used to examine polyglutamine aggregation and toxicity. These fusion constructs do not contain the proline-rich region of the huntingtin exon 1 [52].

### Cultivation procedures

Yeast strains were cultivated using standard media and growth protocols [71] and grown at 30^o^C except when indicated. Complex media contained 2% dextrose (YPD), and synthetic complete media contained the required amino acids and 2% dextrose (SD) or 2% galactose (SGal).

### Curing of BY4741 deletion strains

Pre-existing prions were cured by growing strains on media containing low levels of guanidine hydrochloride (GuHCl), which cures through the inactivation of Hsp104 [72], [73]. Strains were spotted onto YPD plates containing 5mM GuHCl and repeated two to three additional times to ensure that prions were cured.

### Preparation of the *kar1* plasmid donor and introduction of plasmids and prions into deletion strains

Prions and plasmids were introduced into the *kar1* plasmid donor strain. To make a [*PSI* ^+^] *kar1* plasmid donor strain to test for [*PSI* ^+^] maintenance in the deletion strains, the *kar1* plasmid donor strain was crossed to either a weak [*PSI* ^+^] (L1759) or strong [*PSI* ^+^] (L1763) strain containing *Sup35PD-GFP*. *kar1* plasmiductants containing [*PSI* ^+^] were chosen as described in Manogaran *et al*. [48], and confirmed to contain [*PSI* ^+^] by the formation of Sup35PD-GFP aggregates after 16 hours. The [*PSI* ^+^] *kar1* plasmid donor was mated to the BY4741 deletion strains, deletion strain cytoductants were confirmed by testing for auxotrophic markers, and the presence of [*PSI* ^+^] Sup35PD-GFP aggregate formation was examined as described above [54].

To test for the induction of [*PSI* ^+^], prions and plasmids were introduced into the *kar1* plasmid donor strains by crossing to either a [*PIN* ^+^] (L1749 high [*PIN* ^+^]) or [*pin* ^-^] strain (L2910) containing the *Sup35PD-GFP* and *ura3*–*14* plasmids. [*PIN* ^+^] *kar1* plasmid donor strains were confirmed as above and tested for the presence of [*PIN* ^+^] by the formation of Sup35PD-GFP fluorescent rings after 24 hours of induction on copper [54]. Cytoduction of plasmids and prions into the BY4741 library deletion strains has been described previously [48]. To ensure reproducibility, cytoductions were performed in duplicate.

### Induction of [*PSI* ^+^] in cytoduced deletion strains

Cytoduced BY4741 deletion strains, containing [*PIN* ^+^] and both *Sup35PD-GFP* (*HIS3*) and *ura3*–*14* (*LEU2*) plasmids, were spotted onto plasmid selective SD-His-Leu plates plus 50 µM copper sulfate and grown for approximately two days. Strains were resuspended in 300 µL of sterile water and either spotted onto SD-Leu (grown two days) to assess growth, or SD-Leu-Ura (grown at room temperature for five to seven days) to score for [*PSI^+^*] induction. Induction experiments on all 398 yeast deletion library strains were repeated six times to ensure reproducibility. Spots that exhibited no or reduced growth on SD-Leu-Ura, compared to controls, were chosen as candidate genes that affect [*PSI* ^+^] appearance (Table 1).

### Re-engineering of candidate strains

Genetic recombination was used to replace candidate genes (Table 1) with *HIS3* in [*PIN* ^+^] 74-D694. Primers (Table S2), adjacent to sequences flanking the 5′ or 3′ ends of the candidate gene, were used to PCR amplify the *HIS3* gene. PCR products were transformed and His^+^ transformants were confirmed for insertion of the *HIS3* gene in the correct locus. Two to three independent knockout lines (Table S3) were obtained for each deletion, except for *pre9Δ*. To confirm the presence of [*PIN* ^+^], deletion strains were mated to a [*pin^-^*] tester strain containing a *RNQ1-GFP* plasmid. Diploids were checked for the formation of fluorescent Rnq1-GFP aggregates after induction overnight. To test whether *las17Δ, vps5Δ,* or *sac6Δ* maintain [*PSI^+^*], the deletions were cytoduced with [*PSI* ^+^] and checked for growth on –Ade.

### Scoring the induction frequency of [*PSI* ^+^]

Re-engineered deletion strains were transformed with the *Sup35PD-GFP* (*LEU2*) plasmid and grown in SD-Leu plus copper sulfate liquid media for 24 hours. After induction, approximately 10,000 cells were plated on SD–Ade, and a 50-fold dilution of cells was plated on SD+12. Colony counts were obtained from at least one transformant from each independent knock out (see Table S3) on SD+12 vs. SD-Ade. Colony counts from at least three transformants were used to determine the induction frequency.

### Sup35PD-GFP ring formation in re-engineered deletion strains

After 24 hours of induction, cells were examined for the formation of GFP fluorescent rings [54] using a Zeiss Axioskop2 deconvolution workstation equipped with either a X40 Plan-Neofluar or X100 Plan-Apochromat objective lens (Zeiss). Approximately 300 cells were counted from at least one transformant from each independent knock out, for a total of three transformants.

### Determining cell viability of ring-containing cells

Ring containing cells were simultaneously visualized and micromanipulated onto 2% sterile Noble Agar slabs. Slabs were transferred onto YPD media and grown for one to two days.

### Aggregation and toxicity of polyglutamine in re-engineered deletion strains

Strains containing the galactose inducible 25Q or 103Q GFP fusion plasmids were grown overnight in SD-Ura and then washed in water approximately four times to remove residual glucose. To score for aggregation, washed cells were grown in liquid Gal-Ura for one to two hours with shaking and then examined for GFP aggregates. To score for toxicity, log phase uninduced washed cells were serially diluted 20-fold and spotted onto SD-Ura or SGal-Ura.

## Supporting Information

Figure S1 Certain deletion strains have decreased levels of Sup35 and Sup35PD-GFP. [*PIN* ^+^] wildtype or deletion strains containing *Sup35PD-GFP* were grown for 22 hours to an approximate OD_600_ 3.0 at 30°C in the presence of 50 µM copper. All strains are in the 74D-694 background except *sac6Δ*, which is in the BY4741 background. Equivalent amounts of cell lysates were loaded per lane and subjected to SDS-PAGE (see Text S1). Lanes in the left panel were run on the same gel. Blotted proteins were incubated either with anti-GFP antibody, anti-Sup35 C-terminal antibody, or anti-PGK antibody (as indicated).
(0.24 MB TIF)

Figure S2 The [*PIN* ^+^] variant in the deletion strains appears to be unaffected. [*PIN* ^+^] wildtype and deletion strains, containing *Sup35PD-GFP*, were grown for 24 hours in copper media, and equivalent amounts of protein were loaded on a 1.5% agarose gel and subjected to SDD-AGE [53]. Blotted proteins were incubated with an antibody against the Rnq1 protein.
(0.62 MB TIF)

Figure S3 All deletion strains reach saturation after 24 hours of induction, except *las17Δ.* Deletion and wildtype strains containing *Sup35PD-GFP* and [*PIN* ^+^] were grown in plasmid selective media with copper at 30°C. OD_600_ readings were taken at the indicated time points to assess growth.
(0.27 MB TIF)

Figure S4 Vacuole formation is altered in deletion strains, except *hog1Δ*. A. Wildtype and deletion strains were incubated with 5 µM FM4-64 for 20 minutes in YPD, washed and then allowed to incubate for 60 minutes before visualizing internalization of the dye. B. Wildtype cells were treated either with DMSO (gray bars) or various concentrations of Nocodazole (purple bars) during overnight induction of *Sup35PD-GFP*. Percent of rings were calculated after 24 hours of induction. C. Untreated cells (DMSO) or cells treated with 50 ug/ml of Nocodazole from (B) were plated in 20 fold serial dilutions on SD-Ade media to score for [*PSI* ^+^] induction. Growth on SD+Ade indicates there is no growth defect.
(0.98 MB TIF)

Table S1 Candidate deletions screened for [PSI+] induction.
(0.16 MB DOC)

Table S2 Primers used in re-engineering deletion candidates in the 74-D694 genetic background.
(0.05 MB DOC)

Table S3 Re-engineered independent knock out lines in the 74-D694 background used in this study.
(0.05 MB DOC)

Text S1 Supporting information methods.
(0.05 MB DOC)
