# Supplementary figures and images for: Prion Formation and Polyglutamine Aggregation Are Controlled by Two Classes of Genes
Source: PLoS Genet. 2011 May 19;7(5):e1001386. doi: 10.1371/journal.pgen.1001386 (PMC3098188; doi:10.1371/journal.pgen.1001386)

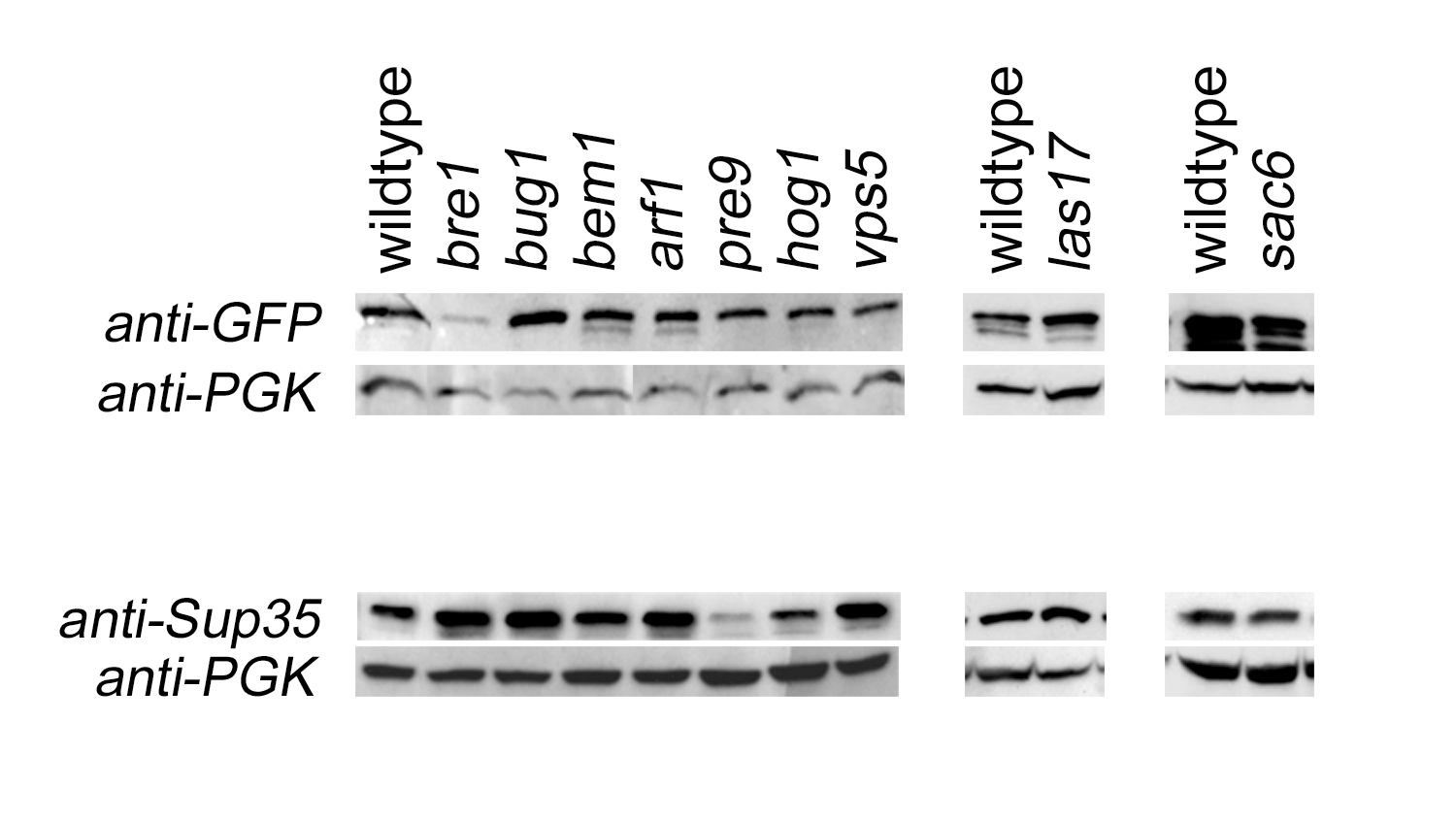

Supplement: Figure S1 — Certain deletion strains have decreased levels of Sup35 and Sup35PD-GFP. [PIN +] wildtype or deletion strains containing Sup35PD-GFP were grown for 22 hours to an approximate OD600 3.0 at 30°C in the presence of 50 µM copper. All strains are in the 74D-694 background except sac6Δ, which is in the BY4741 background. Equivalent amounts of cell lysates were loaded per lane and subjected to SDS-PAGE (see Text S1). Lanes in the left panel were run on the same gel. Blotted proteins were incubated either with anti-GFP antibody, anti-Sup35 C-terminal antibody, or anti-PGK antibody (as indicated). (0.24 MB TIF) [file pgen.1001386.s001.tif]

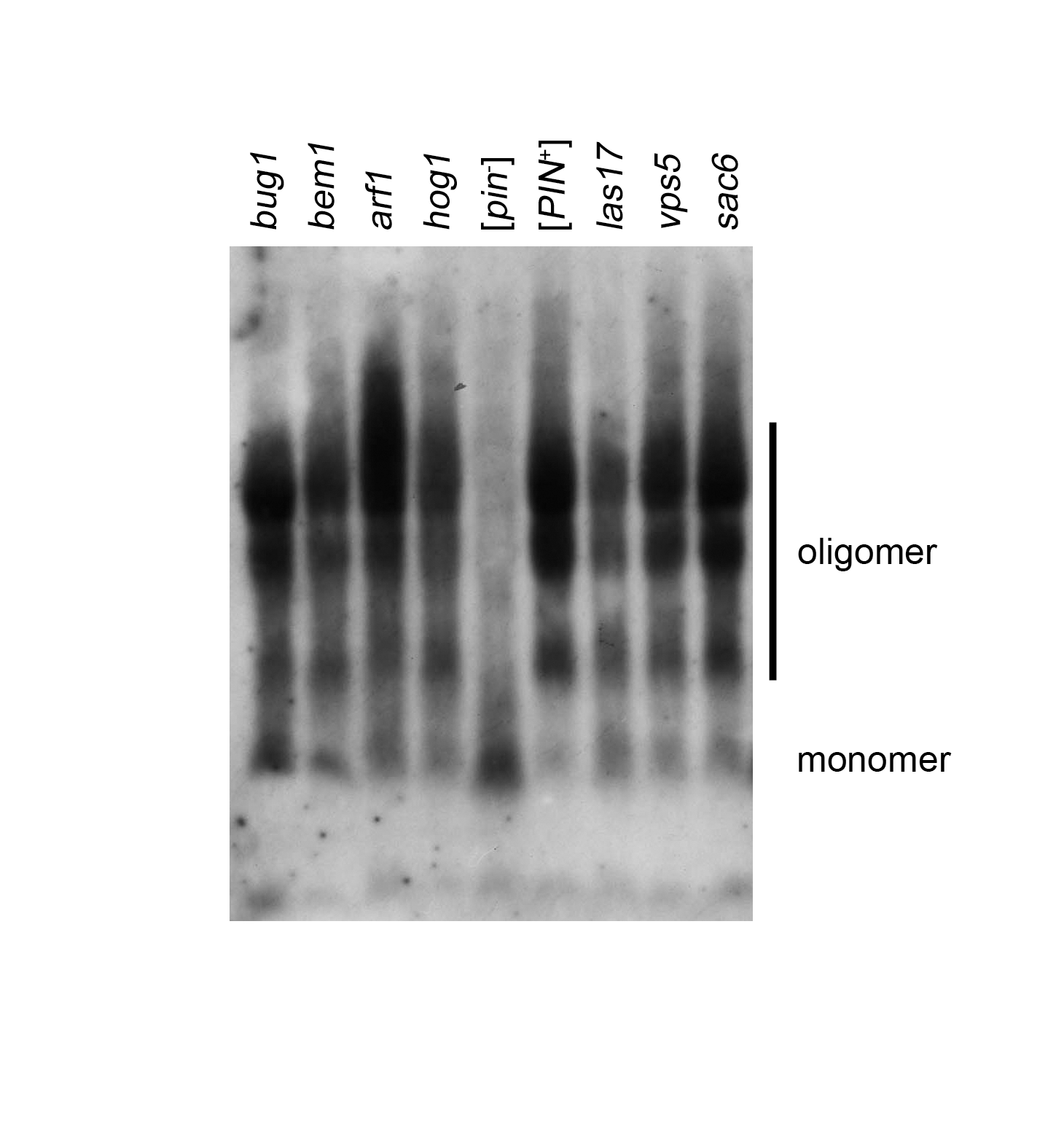

Supplement: Figure S2 — The [PIN +] variant in the deletion strains appears to be unaffected. [PIN +] wildtype and deletion strains, containing Sup35PD-GFP, were grown for 24 hours in copper media, and equivalent amounts of protein were loaded on a 1.5% agarose gel and subjected to SDD-AGE [53]. Blotted proteins were incubated with an antibody against the Rnq1 protein. (0.62 MB TIF) [file pgen.1001386.s002.tif]

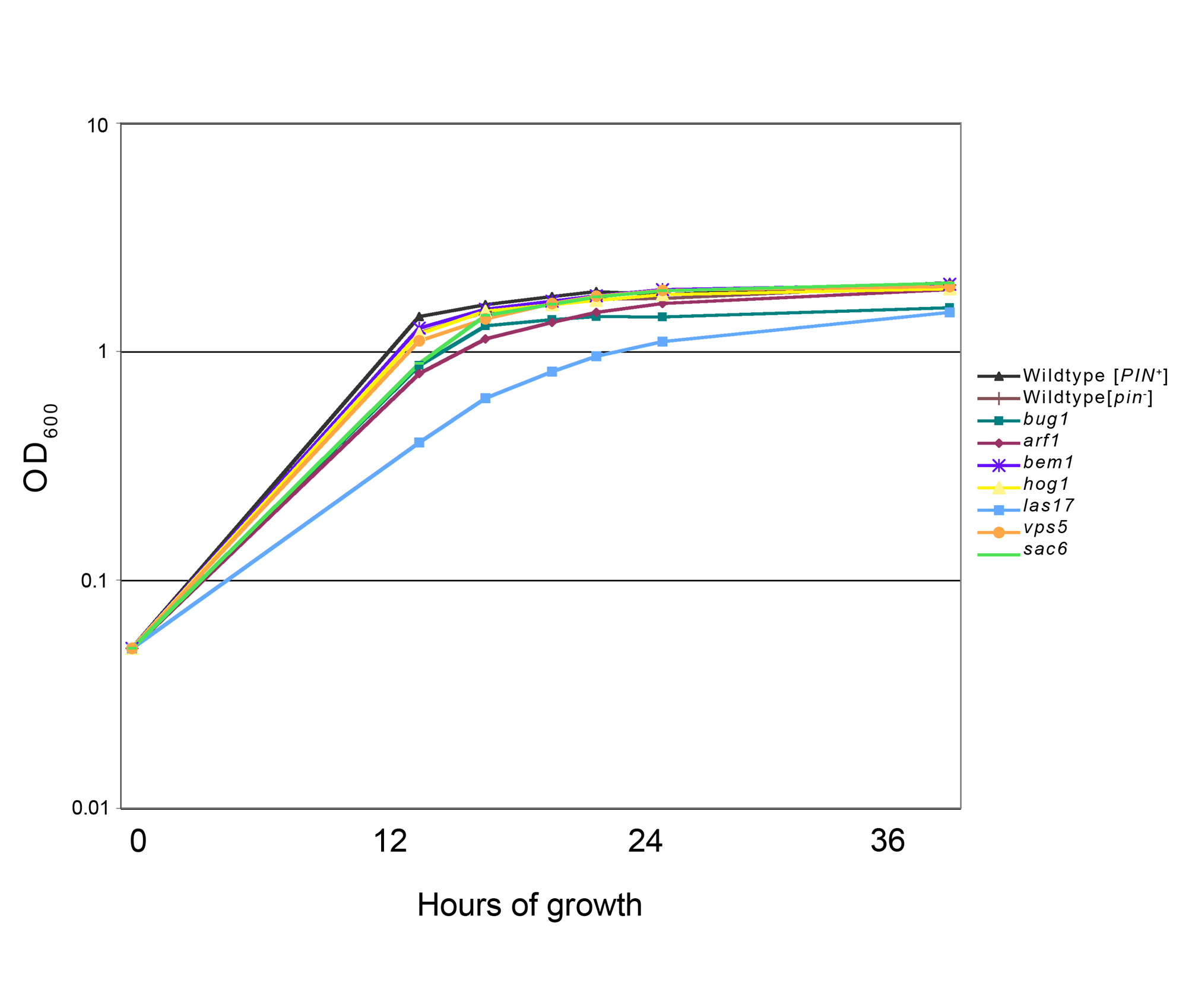

Supplement: Figure S3 — All deletion strains reach saturation after 24 hours of induction, except las17Δ. Deletion and wildtype strains containing Sup35PD-GFP and [PIN +] were grown in plasmid selective media with copper at 30°C. OD600 readings were taken at the indicated time points to assess growth. (0.27 MB TIF) [file pgen.1001386.s003.tif]

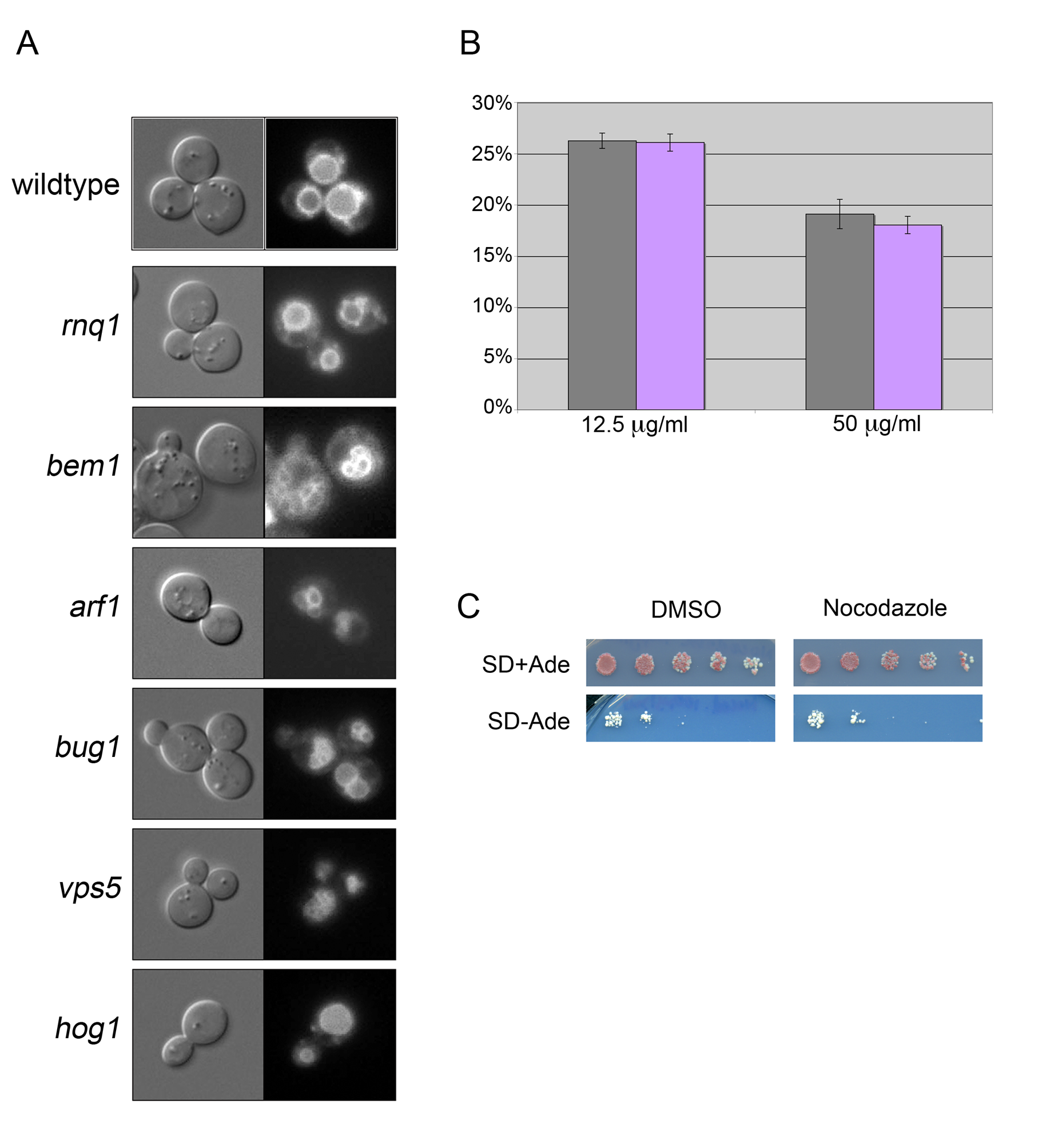

Supplement: Figure S4 — Vacuole formation is altered in deletion strains, except hog1Δ. A. Wildtype and deletion strains were incubated with 5 µM FM4-64 for 20 minutes in YPD, washed and then allowed to incubate for 60 minutes before visualizing internalization of the dye. B. Wildtype cells were treated either with DMSO (gray bars) or various concentrations of Nocodazole (purple bars) during overnight induction of Sup35PD-GFP. Percent of rings were calculated after 24 hours of induction. C. Untreated cells (DMSO) or cells treated with 50 ug/ml of Nocodazole from (B) were plated in 20 fold serial dilutions on SD-Ade media to score for [PSI +] induction. Growth on SD+Ade indicates there is no growth defect. (0.98 MB TIF) [file pgen.1001386.s004.tif]
