# Supplementary material for: Prion Formation and Polyglutamine Aggregation Are Controlled by Two Classes of Genes
Source: PLoS Genet. 2011 May 19;7(5):e1001386. doi: 10.1371/journal.pgen.1001386 (PMC3098188; doi:10.1371/journal.pgen.1001386)
Supplement: Table S1 — Candidate deletions screened for [PSI+] induction. (0.16 MB DOC) [file pgen.1001386.s005.doc]

**Supplementary Table 1. Candidate deletions screened for [*PSI*^+^] induction.**

| Reduced Toxicity Candidates  (unknown)=function unknown | | |
| --- | --- | --- |
| YAL061W (BDH2) | YAL055W (PEX22) | YAL053W (FLC2) |
| YAL046C (unknown) | YAL040C (CLN3)* | YAL031C (FUN21) |
| YAL011W (SWC3) | YLL013C (PUF3) | YLL026W (HSP104) |
| YLL055W (YCT1) | YLR085C (ARP6) | YML061C (PIF1) |
| YML035C-A (unknown) | YML033W (SRC1) | YMR029C (FAR8) |
| YMR182C (RGM1) | YMR193W (MRPL24) | YNL047C (SLM2) |
| YNL259C (ATX1) | YOR014W (RTS1) | YOR042W (CUE5) |
| YOR058C (ASE1) | YOR080W (DIA2) | YOR312C (RPL20B) |
| YOL015W (IRC10) | YOL045W (PSK2) | YOL057W (unknown) |
| YPL195W (APL5) | YPL177C (CUP9) | YBR177C (EHT1)* |
| YBR200W (BEM1)* | YBR213W (MET8) | YDR068W (DOS2) |
| YDR096W (GIS1) | YDR098C (GRX3) | YDR101C (ARX1) |
| YDR112W (ICR2) | YDR383C (NKP1) | YDR430C (CYM1) |
| YDR436W (PPZ2) | YER011W (TIR1) | YER047C (SAP1) |
| YER048C (CAJ1) | YGR125W (unknown) | YGR138C (TPO2) |
| YGR152C (RSR1) | YHR038W (RRF1) | YHR114W (BZZ1) |
| YHR163W (SOL3) | YCL005W (LDB16) | YCL028W (RNQ1) |
| YKL054C (DEF1) | YKL063C (unknown) | YKL079W (SMY1) |
| YKL090W (CUE2) | YOR127W (RGA1) | YOR138C (RUP1) |
| YOR161C (PNS1) | YOR198C (BFR1) | YOR288C (MPD1) |
| YJL207C (LAA1) | YLR412W (unknown) | YLR238W (FAR10) |
| YLR278C (unknown) | YLR292C (SEC72) | YLR295C (ATP14) |
| YDR203W (unknown) | YGL200C (EMP24) | YGL203C (KEX1) |
| YGL217C (unknown) | YGL229C (SAP4) | YGL252C (RTG2) |
| YGR011W (unknown) | YGR018C (unknown) | YGR022C (unknown) |
| YGR025W (unknown) | YPL030W (unknown) | YPR132W (RPS23b) |
| YPR167C (MET16) | YDR525W-A (SNA2) | YBL027W (RPL19B) |
| YPL158C (unknown) | YGL033W (HOP2) | YGL067W (NPY1) |
| YNL199C (GCR2) | YNL175C (NOP13) | YDR309C (GIC2) |
| YNL198C (unknown) | YIL074C (SER33) | YFR008W (FAR7) |
| YIL084C (SDS3) | YMR062C (ECM40) | YMR065W (KAR5) |
| YIR017C (MET28) | YMR069W (NAT4) | YMR087W (unknown) |
| YMR068W (AVO2) | YML107C (PML39) | YMR106C (YKU80) |
| YMR088C (VBA1) | YOL162W (unknown) | YJR115W (unknown) |
| YJL080C (SCP160) | YLR445W (unknown) | YML010C-B (unknown) |
| YLR437C (unknown) | YDL243C (AAD4) | YCR043C (unknown) |
| YDL192W (ARF1)* | YJL020C (BBC1) | YJL024C (APS3) |
| YJL007C (unknown) | YJR010W (MET3) | YJR051W (OSM1) |
| YJR010C-A (SPC1) | YJR062C (NTA1) | YDL100C (ARR4) |
| YJR053W (BFA1) | YDL117W (CYK3) | YDL188C (PPH22) |
| YDL109C (unknown) | YNR032W (PPG1) | YNR049C (MSO1) |
| YNL016W (PUB1) | YBR010W (HHT1) | YBR012C (unknown) |
| YBL106C (SRO77) | YBR068C (BAP2) | YNL139C (RLR1) |
| YBR016W (unknown) | YIL146C (ECM37) | YIL159W (BNR1) |
| YNL127W (FAR11) | YIR009W (MSL1) | YDR202C (RAV2) |
| YIR003W (unknown) | YBR288C (APM3) | YMR138W (CIN4) |
| YGR063C (SPT4) | YFR030W (MET10) | YBR090C-A (NHP6B) |
| YOR298C-A (MBF1) | YBR119W (MUD1) | YBR156C (SLI15) |
| YBR093C (PHO5) | YGL132W (unknown) | YER114C (BOI2) |
| YDL088C (ASM4) | YMR052W (FAR3) | YBR075W (unknown) |
| YER124C (DSE1) |  |  |

| Enhanced Toxicity Candidates | | |
| --- | --- | --- |
| YAL023C (PMT2) | YAL021C (CCR4) | YAR002W (NUP60) |
| YAR003W (SWD1) | YAR015W (ADE1) | YLL029W (unknown) |
| YLL039C (UBI4) | YLR079W (SIC1) | YLR113W (HOG1) |
| YMR186W (HSC82) | YMR198W (CIK1) | YMR223W (UBP8) |
| YMR224C (MRE11) | YMR294W (JNM1) | YNL329C (PEX6) |
| YNL322C (KRE1) | YNL311C (unknown) | YNL299W (TRF5) |
| YNL291C (MID1) | YOR008C (SLG1) | YOR043W (WHI2) |
| YOR070C (GYP1)* | YOR089C (VPS21) | YOR360C (PDE2) |
| YOL018C (TLG2)* | YOL076W (MDM20) | YPL250C (ICY2) |
| YPL226W (NEW1) | YPL198W (RPL7B) | YPL179W (PPQ1) |
| YPL165C (SET6) | YPL150W (unknown)* | YPL149W (ATG5) |
| YPL145C (KES1) | YPL139C (UME1) | YPL133C (RDS2) |
| YPL130W (SPO19) | YPL101W (ELP4) | YBR175W (SWD3) |
| YEL013W (VAC8)* | YEL042W (GDA4) | YER020W (GPA2) |
| YHL047C (ARN2) | YHL031C (GOS1)* | YHL028W (WSC4) |
| YHL019C (APM2) | YHL013C (OTU2) | YHL007C (STE20) |
| YHR015W (MIP6) | YHR079C (IRE1) | YHR081W (LRP1) |
| YHR087W (unknown) | YHR104W (GRE3) | YHR109W (CTM1) |
| YHR110W (ERP5) | YHR121W (LSM12) | YHR123W (EPT1) |
| YHR124W (NDT80) | YHR135C (YCK1) | YHR200W (RPN10) |
| YCL032W (STE50) | YLR177W (unknown) | YLR211C (unknown) |
| YLR216C (CPR6) | YGR043C (unknown) | YGR045C (unknown) |
| YGR055W (MUP1) | YGR057C (LST7) | YGR081C (SLX9) |
| YGR085C (RPL11B) | YGR111W (unknown) | YGR121C (MEP1) |
| YOR113W (AZF1) | YOR132W (VPS17)* | YOR140W (SFL1) |
| YOR182C (RPS30B) | YOR183W (FYV12) | YOR191W (RIS1) |
| YJL204C (RCY1) | YJL190C (RPS22A) | YJL187C (SWE1) |
| YJL168C (SET2) | YJL164C (TPK1) | YJL144W (unknown) |
| YJL139C (YUR1) | YJL135W (unknown) | YJL134W (LCB3) |
| YLR354C (TAL1) | YLR373C (VID22)* | YLR402W (unknown) |
| YLR407W (unknown) | YLR235C (unknown) | YLR263W (RED1) |
| YDR200C (VPS64)* | YDR207C (UME6) | YGL197W (MDS3) |
| YGL235W (unknown) | YGL237C (HAP2) | YPL089C (RLM1) |
| YPL074W (YTA6) | YPL069C (BTS1) | YPL061W (ALD6) |
| YPL047W (SGF11) | YPL002C (SNF8) | YPR120C (CLB5) |
| YPR164W (MMS1) | YPR170C (unknown) | YPR188C (MLC2) |
| YPR197C (unknown) | YFR036W (CDC26) | YBL047C (EDE1) |
| YGL027C (CWH41) | YGL066W (SGF73) | YNL191W (DUG3) |
| YNL183C (NPR1) | YNL148C (ALF1) | YKL211C (TRP3) |
| YKL212W (SAC1) | YKR021W (ALY1) | YKR026C (GCN3) |
| YKR030W (GMH1) | YDR293C (SSD1) | YDR320C (SWA2) |
| YIL013C (PDR11) | YIL017C (VID28) | YIL028W (unknown) |
| YIL029C (unknown) | YIL041W (GVP36) | YIL053W (RHR2) |
| YIL073C (SPO22) | YIL090W (ICE2) | YIL007C (NAS2) |
| YIL038C (NOT3) | YIL042C (PKP2) | YIL052C (RPL34b) |
| YIL092W (unknown) | YFL031W (HAC1) | YFL032W (unknown) |
| YFR031C-A (RPL2A) | YGR270W (YTA7) | YNR070W (unknown) |
| YOL101C (IZH4) | YOL105C (WSC3) | YOL117W (RRI2) |
| YOL119C (MCH4) | YER091C (MET6) | YHR017W (YSC83) |
| YML097C (VPS9) | YML102W (CAC2) | YMR116C (ASC1) |
| YPR065W (ROX1) | YPR070W (MED1) | YPR096C (unknown) |
| YPR098C (unknown) | YJL123C (unknown) | YJL095W (BCK1)* |
| YJL093C (TOK1) | YJL062W (LAS21) | YEL012W (UBC8) |
| YLL007C (unknown) | YOL159C (unknown) | YJR075W (HOC1) |
| YJR079W (unknown) | YJR083C (ACF4) | YJR088C (unknown) |
| YJR110W (YMR1) | YJR129C (unknown) | YKR091W (SRL3) |
| YLR435W (TSR2) | YLR452C (SST2) | YML021C (UNG1) |
| YMR174C (PAI3) | YNR051C (BRE5) | YNR059W (MNT4) |
| YGL235W (unknown) | YJR118C (ILM1) | YJR140C (HIR3) |
| YML068W (ITT1) | YDR024W (FYV1) | YCR034W (FEN1)* |
| YDL099W (BUG1) | YDL116W (NUP84) | YDL183C (unknown) |
| YNL023C (FAP1) | YNL040W (unknown) | YNL041C (COG6) |
| YNR013C (PHO91) | YNR031C (SSK2) | YNR047W (unknown) |
| YBR021W (FUR4) | YNL136W (EAF7) | YNL133C (FYV6) |
| YNL069C (RPL16B) | YIL110W (unknown) | YIL168W (SDL1) |
| YIR001C (SGN1) | YIR016W (unknown) | YDR007W (TRP1) |
| YDR048C (unknown) | YNL068C (FKH2) | YNL074C (MLF3) |
| YNL082W (PMS1) | YNL095C (unknown) | YBR189W (RPS9B) |
| YDL133C-A (RPL41B) | YDR537C (unknown) | YGR040W (KSS1) |
| YGR050C (unknown) | YGR250C (unknown) | YKR010C (TOF2) |
| YKR036C (CAF4) | YCR048W (RPL21A) | YDR071C (PAA1) |
| YLR246W (ERF2) | YLR346C (unknown) | YLR370C (ARC18)* |
| YLR394W (CST9) | YML027W (YOX1) | YMR139W (RIM11) |
| YFR019W (FAB1) | YMR037C (MSN2) | YNR052C (POP2) |
| YBR111C (YSA1) | YBR171W (SEC66) | YDL032W (unknown) |
| YDL081C (RPP1A) | YDR448W (ADA2) | YDR469W (SDC1) |
| YDR500C (RPL37B) | YGL144C (ROG1) | YER115C (SPR6) |
| YER128W (unknown) | YDL074C (BRE1) | YHR028C (DAP2) |
| YHR033W (unknown) | YFL001W (DEG1) | YGR289C (MAL11) |
| YFL033C (RIM15)* |  |  |
| Other candidates scored for [PSI^+^] induction | | |
| YNL107W (YAF9) | YGR135W (PRE9) | YNR031C (SSK2) |
| YMR048W (CSM3) | YKL213C (DOA1) | YFR010W (UBP6) |
| YBR201W (DER1) | YCR059C (YIH1) | YIL156W (UBP7) |

* designated vacuole fragmentation mutant as described by Seeley *et al*. [66].
