# Supplementary material for: Prion Formation and Polyglutamine Aggregation Are Controlled by Two Classes of Genes
Source: PLoS Genet. 2011 May 19;7(5):e1001386. doi: 10.1371/journal.pgen.1001386 (PMC3098188; doi:10.1371/journal.pgen.1001386)
Supplement: Table S2 — Primers used in re-engineering deletion candidates in the 74-D694 genetic background. (0.05 MB DOC) [file pgen.1001386.s006.doc]

**Supplementary Table 2. Primers used in re-engineering deletion candidates in the 74-D694 genetic background.**

| Gene name | Primer 1 | Primer 2 |
| --- | --- | --- |
| *bem1::HIS3* | AAGCACTGTGTGAAAAGAAATTGTCAAGAAAGCCATATAAATGACAGAGCAGAAAGCCCTAGTAAAGC | CAAGTAAAGAAGAAAAATGCTTCGTCTTCTAACACTAGATCTACATAAGAACACCTTTGGTGG |
| *rnq1::HIS3* | TTTTGTTTGGTCGTTTTCTCAATATAATCTACATCATCATATGACAGAGCAGAAAGCCCTAGTAAAGC | CCATTTCGTTTTTTATGTGGGAGGTTCTACTTCTCCCTTACTACATAAGAACACCTTTGGTGG |
| *scp160::HIS3* | TAAAATATACTTCCCACACCCCCTCCTTCCATTATAACTGATGACAGAGCAGAAAGCCCTAGTAAAGC | AAAGCCAAAATCTATATTGAAAAAAATTGGTTTCAAAGAGCTACATAAGAACACCTTTGGTGG |
| *def1::HIS3* | TTTTGTTTGGTCGTTTTCTCAATATAATCTACATCATCATATGACAGAGCAGAAAGCCCTAGTAAAGC | CCATTTCGTTTTTTATGTGGGAGGTTCTACTTCTCCCTTACTACATAAGAACACCTTTGGTGG |
| *pre9::HIS3* | TTTAATAATTGATTATTGGATATAGTTAGTAGTGTTAAACATGACAGAGCAGAAAGCCCTAGTAAAGC | TGCGTACATATTTATATAAGCATGAAGTCAAACAATACTTCTACATAAGAACACCTTTGGTGG |
| *rpp1a::HIS3* | GTCCAATACAACAGCTTAAACCGATTATCTCTAAAATAACATGACAGAGCAGAAAGCCCTAGTAAAGC | ATGTTAGAATATGCAATTTCTTCTAAACAGTGCGGCACTTCTACATAAGAACACCTTTGGTGG |
| *bfr1::HIS3* | TCAACGTAATAGCATATTTTCTAACAACACAGCCATTGCCATGACAGAGCAGAAAGCCCTAGTAAAGC | CCTCAACCAAAGAAAAATTAAGTAATGAAGAAAGATCAGGCTACATAAGAACACCTTTGGTGG |
| *rpl20b::HIS3* | AGATAATAGAAGAGAACCGTAACAAAGGAATCAAGCAAAGATGACAGAGCAGAAAGCCCTAGTAAAGC | CAAAATTTTATTATACTAAAAAACGTAGAAATATCTTGTTGGCCTACATAAGAACACCTTTGGTGG |
| *bre1::HIS3* | ACCGTTTTTATGCTAATCGTGCTAGCTGATAATAATCAGAATGACAGAGCAGAAAGCCCTAGTAAAGC | GGATATAACACAAACAGTGGAAAAGTGGTAGAATAATTAGCTACATAAGAACACCTTTGGTGG |
| *hog1::HIS3* | ACAAAGGGAAAACAGGGAAAACTACAACTATCGTATATAAATGACAGAGCAGAAAGCCCTAGTAAAGC | AGAAGTAAGAATGAGTGGTTAGGGACATTAAAAAAACACGCTACATAAGAACACCTTTGGTGG |
| *ase1::HIS3* | CTGGCATTAGAATTCAAAGGTTTCATTTTGGATTCACTACATGACAGAGCAGAAAGCCCTAGTAAAGC | GAAAGAAAGAGACAATGTAGCGAAGGCTAGAAAGTGATGTGCTACATAAGAACACCTTTGGTGG |
| *arf1::HIS3* | GCATTGAAGGTATAAGAAAGAACTCAAACAGGTTTAATAGATGACAGAGCAGAAAGCCCTAGTAAAGC | TGTTTCATTTAGTTTATACAAGCGTATTTGATCCATATTCCTACATAAGAACACCTTTGGTGG |
| *bug1::HIS3* | TTGAACCAACCTGAATTGAGCAAGTTTATCATATAGTTACATGACAGAGCAGAAAGCCCTAGTAAAGC | TAAACCCTTAATTAAAATATATACGCGAAAATATTCATTCCTACATAAGAACACCTTTGGTGG |
| *cik1::HIS3* | TTTGGCATTTGAAACTCGTTGACATAAGCTTTATTATTAGATGACAGAGCAGAAAGCCCTAGTAAAGC | AAGGGAACGAGACGAAGTGATTGTAGCGCACAAGATCTGGCTACATAAGAACACCTTTGGTGG |
| *lst7::HIS3* | CTCCATAAAATAAGTTAGCGTTTAAATGGTTGATGAAATAGATGACAGAGCAGAAAGCCCTAGTAAAGC | ATCGGTTATTAGGTTACCAGCATTTATCTCAGTTTATTTGCTACATAAGAACACCTTTGGTGG |
| *nas2::HIS3* | TTATCACCAGAGTAAAAAGGAGCAAGAATTAGAATAAGAACATGACAGAGCAGAAAGCCCTAGTAAAGC | ATGCAGATAGTATAAAAAGTAATAGTAATAACTGGATTGGCTACATAAGAACACCTTTGGTGG |
| *swa2::HIS3^*^* | CTTCTGGAAAGGACGCAGCCTGCAAGAAACAGTCAACATCATGACAGAGCAGAAAGCCCTAGTAAAGC | AGTACATATCAAAAACAACTGAGCGAAGCAGGCACACAAGCTACATAAGAACACCTTTGGTGG |
| *trf5::HIS3* | TTTCAAATAAACAAACGAGGGCGGAGTTTATTGGGTCGTCATGACAGAGCAGAAAGCCCTAGTAAAGC | TCTTGTATAAATAGTAAATAGTCTATAAGAGTCTATATTGTGCTACATAAGAACACCTTTGGTGG |
| *ydr048c::HIS3* | CTATTTGGAGATCTCCAGACTCCTTGTTTCTATCATTATCATGACAGAGCAGAAAGCCCTAGTAAAGC | CCGCTACATTGTTAATTTCGGTCACGGTACCCACCCTTTCCTACATAAGAACACCTTTGGTGG |
| *his3Δ200::HIS3* | AGAGCTTGGTGAGCGCTAGG | TATTTTTTTTCTCGAGTTCAAGAGAAAAAAAAAGAAAAAG |
| *vps5::HIS3* | CAGCAGGGATTTTATAAACTTTCATACATCCTGCAATAACATGACAGAGCAGAAAGCCCTAGTAAAGC | AATTCATAAATCCTGAGGAACGTGACACATAAAGTTATTGCTACATAAGAACACCTTTGGTGG |
| *las17::HIS3* | CCGCTACAAATTACAGTTCGTTACTTTAAGTGTTGATAGGATGACAGAGCAGAAAGCCCTAGTAAAGC | TTTTCTATAACAGTAGTTTCATCTTTGTTTGCATTCC CTACATAAGAACACCTTTGGTGG |
| *sac6::HIS3* | AGAAGCTGATATATTAGCCCTAAGGAGTACACCAAAACACATGACAGAGCAGAAAGCCCTAGTAAAGC | AAGCTGAGTAGAAAACAGGTTACGAAAGTTGTTTGTTGGCCTACATAAGAACACCTTTGGTGG |
