# Supplementary material for: Prion Formation and Polyglutamine Aggregation Are Controlled by Two Classes of Genes
Source: PLoS Genet. 2011 May 19;7(5):e1001386. doi: 10.1371/journal.pgen.1001386 (PMC3098188; doi:10.1371/journal.pgen.1001386)
Supplement: Table S3 — Re-engineered independent knock out lines in the 74-D694 background used in this study. (0.05 MB DOC) [file pgen.1001386.s007.doc]

**Supplementary Table 3. Re-engineered independent knock out lines in the 74-D694 background used in this study.**

| Deletion | Strain number |
| --- | --- |
| *bug1*Δ | M208  M209  M210 |
| *bem1*Δ | M157  M158  M159 |
| *arf1*Δ | M199  M200 |
| *hog1*Δ | M183  M184 |
| *las17*Δ | M173  M175 |
| *vps5*Δ | M176  M177 |
| *sac6*Δ | L3129  L3130  L3131 |
| *pre9*Δ | M163 |
| *bre1*Δ | M180  M181 |

Multiple independent lines were obtained for each deletion strain, except for *pre9Δ*. Transformants from each independent line were used in the experiments in this study. In the case where there were only two independent deletions, multiple transformants were tested from each of the independent lines to calculate standard error for [*PSI*^+^] induction frequency (Figure 2), ring formation (Figure 3A), viability (Figure 3B) and polyglutamine aggregation (Figure 4B).
