# Supplementary material for: Prion Formation and Polyglutamine Aggregation Are Controlled by Two Classes of Genes
Source: PLoS Genet. 2011 May 19;7(5):e1001386. doi: 10.1371/journal.pgen.1001386 (PMC3098188; doi:10.1371/journal.pgen.1001386)
Supplement: Text S1 — Supporting information methods. (0.05 MB DOC) [file pgen.1001386.s008.doc]

**Supporting Information**

**Methods**

**Analysis of protein levels found in re-engineered candidate strains**

After 24 hours of induction, cells were harvested and crude cell extracts were mechanically lysed using glass beads in 600 μL of lysis buffer (50mM Tris-HCl, pH 7.5, 50 mM KCl, 10 mM MgCl_2_ and 5% glycerol), supplemented with protease inhibitor cocktail (Sigma) and 10 mM PMSF. Cells were vortexed for 30 seconds, allowed to incubate on ice for 30 seconds (repeated five times) and lysates were precleared at 600 X *g* for one minute. Protein concentrations were determined using Bio-rad protein reagent. Equivalent concentrations of protein were added to loading buffer (2% SDS, 25 mM Tris-HCl, 200 mM glycine, 5% glycerol and 0.8% β-mercaptomethanol), boiled for ten minutes and run on Bio-Rad Tris-glycine ready gels for SDS-PAGE. Standard Western Blot procedures were performed using anti-Rnq1 antibody.

**Analysis of SDS-resistant oligmers**

Unboiled protein samples, prepared as described above, were incubated at room temperature for seven minutes and loaded onto 1.5% agarose gels. Proteins were blotted onto PVDF membrane, which were then subjected to standard Western blot procedures using antibodies against Sup35, GFP or Pgk1 proteins [83], as indicated.

**FM4-64 treatment**

Cells were grown in liquid YPD to log phase, and then washed and incubated with 8μM FM4-64 (Molecular Probes) in liquid YPD. Cells were visualized using a Texas Red filter after 30 and 60 minutes of incubation.

**Nocodazole treatment**

Wild type cells were grown in –Leu media plus 50 μM copper supplemented with either 12.5 μg/ml or 50 μg/ml of Nocodazole. After 24 hours of induction, the number of rings found in treated and untreated cells were determined, and 10 fold serial dilutions of cells were spotted on either SD+12 or SD-Ade media to score for [*PSI*^+^] induction.
